# The effects of remimazolam on emergence agitation in patients undergoing nasal surgery: a clinical randomized controlled trial

**DOI:** 10.7717/peerj.21018

**Published:** 2026-03-19

**Authors:** Xuelei Zhou, Xianchun Liu, Linlin Chen, Li Zhao, Wei Mao, Longyi Zhang, Ying Xie, Linji Li

**Affiliations:** 1 Department of Anesthesiology, Beijing Anzhen Nanchong Hospital, Capital Medical University & Nanchong Central Hospital, The Second Clinical Medical College, North Sichuan Medical College, Nanchong, Sichuan, China; 2 Department of Anesthesiology, Meishan City People’s Hospital, Meishan, Sichuan, China

**Keywords:** Remimazolam, Emergence agitation, Nasal surgery, Propofol, Adverse effects

## Abstract

**Background:**

Emergence Agitation (EA) is a common postoperative complication, particularly prevalent following nasal surgeries. The occurrence of EA not only elevates the risk of self-inflicted injuries or harm to others but may also lead to a series of severe postoperative complications. This study aims to evaluate the effect of remimazolam on EA in patients undergoing nasal surgery and compare its efficacy with that of conventional anesthetic agents.

**Materials and methods:**

This randomized controlled trial included 100 patients undergoing elective nasal surgery under general anesthesia from June to September 2024. Patients were randomly assigned (1:1) to the R group (remimazolam) or the C group (propofol). Agitation levels were measured using Riker Sedation-Agitation Scale (SAS), recording each patient’s highest score. The primary outcome was the incidence of EA, with secondary outcomes including hemodynamic parameters, emergence time, and adverse events.

**Results:**

The results indicated that the incidence of EA was significantly lower in the R group compared to the C group (22% *vs.* 49%; Risk Difference −27% (95% CI [−50% to −0.3%]); Relative Risk 0.45 (95% CI [0.25–0.81]); *P* = 0.005). Patients in the remimazolam group also demonstrated more stable hemodynamics, with a lower incidence of hypotension (18% *vs.* 53%, *P* < 0.001) and significantly reduced occurrence of injection pain (2% *vs.* 41%, *P* < 0.001). However, there were no significant differences between the two groups in terms of adverse effects such as bradycardia, postoperative wound bleeding, and postoperative nausea and vomiting.

**Conclusions:**

Remimazolam holds significant clinical application value and potential in surgeries with a high incidence of EA.

## Introduction

Emergence agitation (EA), first identified and described in the 1970s, has been defined as a transient, self-limiting, and non-fluctuating state of psychomotor excitement (Safavynia et al., 2018). In the state of EA, patients may engage in high-risk behaviors that pose a danger to themselves and others, such as removing catheters, causing wound dehiscence, falling out of bed, or even attacking healthcare personnel. Moreover, EA can precipitate a range of severe complications, including postoperative delirium, hypoxia, aspiration pneumonia, hemorrhage, the need for reoperation, and cardiovascular events (Hahn et al., 2013; Lepousé et al., 2006). Critically, EA to extended hospital stays, higher morbidity, and increased mortality rates and healthcare costs (Fields et al., 2018; Marcantonio, 2017).

The precise pathophysiology of EA remains largely unclear, but it is particularly noteworthy that patients undergoing otolaryngologic surgeries are at a significantly higher risk for EA, with the incidence following nasal surgery reported to be as high as 55.4% (Demir & Yuzkat, 2018; Lee et al., 2019; Wang et al., 2025; Wegner et al., 2025b; Yu et al., 2010). This phenomenon may be closely linked to contributing factors such as nasal trauma, postoperative airway contamination, sensations of suffocation, and nasal obstruction (Eckenhoff, Kneale & Dripps, 1961).

Remimazolam is a novel ultra-short-acting benzodiazepine, primarily metabolized by non-specific plasma esterases. This unique metabolic mechanism provides several advantages in clinical applications, including rapid onset, strong reversibility, predictable duration of action, and no accumulation effects (Schüttler et al., 2020; Sneyd, Gambus & Rigby-Jones, 2021; Stöhr et al., 2021). Current research indicates that remimazolam offers more stable hemodynamic properties during anesthesia induction and maintenance compared to propofol, while maintaining comparable anesthesia efficacy and postoperative recovery quality (Chae et al., 2022; Choi et al., 2022). However, the impact of benzodiazepines on the incidence of EA remains a subject of debate in the literature (Chandler et al., 2013; Zhang et al., 2013). Nevertheless, benzodiazepines have already been widely utilized in the treatment of EA (Hui, 2018; Kawai et al., 2019; Kim et al., 2021). For instance, studies have shown that midazolam and remimazolam are as effective as dexmedetomidine in treating EA (Deng et al., 2022; Kurhekar et al., 2018). Furthermore, a recent study involving elderly patients undergoing hip replacement surgery reported that remimazolam significantly reduced the incidence of postoperative agitation (Duan et al., 2023). Therefore, in this study, we aimed to investigate if intravenous remimazolam can reduce the incidence of EA in nasal surgery patients.

## Materials & Methods

### Study design

This study is a randomized controlled single-blind study and was conducted as a single-center study at Nanchong Central Hospital in Nanchong, Sichuan, China. It has been registered in the Chinese Clinical Trial Registry (registration number: ChiCTR2400085806, 19/06/2024), and ethical approval was obtained from the Ethical Review Board of Nanchong Central Hospital (Ethical Approval No: 2024063), Sichuan, China. All participants, or their authorized representatives, provided written informed consent prior to surgery. The study was conducted in strict accordance with the ethical principles outlined in the Helsinki Declaration.

### Sample size calculation

According to previous studies, the incidence of EA following otolaryngology surgery is approximately 55.4% (Yu et al., 2010). Remimazolam decreased the incidence of emergence agitation (EA) in elderly patients undergoing hip replacement surgery (10% *vs.* 33%) (Duan et al., 2023), and also significantly reduced the incidence of EA in patients undergoing tonsillectomy and adenoidectomy (12% *vs.* 44%) (Yang et al., 2022). Additionally, a pilot study conducted in our research observed a significant reduction in the incidence of EA with remimazolam administration. Based on these findings and previous evidence (Deng, Qin & Wu, 2022; Duan et al., 2023; Kim et al., 2013; Yang et al., 2022), we hypothesize that remimazolam can reduce the incidence of EA by 50%. The required sample size was calculated using an alpha level (*α*) of 0.05 and a power of 80%, yielding a total of 46 subjects per group, with 50 subjects per group included to account for potential dropouts.

### Study population

A total of 100 patients undergoing elective nasal surgery under general anesthesia between June and September 2024 were included. The types of nasal surgeries included septoplasty, sinus surgery, turbinate surgery, or combined surgeries.

Inclusion Criteria: Patients undergoing elective nasal surgery under general anesthesia, of any gender, aged ≥18 years; Body mass index (BMI) between 19 and 28 kg/m^2^; American Society of Anesthesiologists (ASA) physical status classification of I-III; Participants must provide signed informed consent agreeing to take part in the study.

Exclusion Criteria: Patients with known allergies to the study drugs; Acute respiratory infections, acute exacerbation of chronic obstructive pulmonary disease (COPD), poorly controlled asthma or diabetes mellitus (both type 1 and type 2); Psychiatric disorders, including schizophrenia, depression, cognitive impairment, etc.; Patients with uncontrolled hypertension (systolic blood pressure (SBP) >160 mmHg or diastolic blood pressure (DBP) >100 mmHg), or heart rate (HR) <60 bpm or >100 bpm; Pregnant or lactating women; Patients with severe comorbidities.

Withdrawal Criteria: Patients were withdrawn from the study if there was a change in the surgical procedure or if any unexpected adverse events occurred.

### Randomization and blinding

This study involved patients undergoing elective nasal surgery under general anesthesia. Patients were randomly assigned (1:1) to the R or C group using a computer-generated random number table by an independent researcher. Although anesthesiologists were aware of the group assignments due to the distinct appearance of the drugs, they were not involved in data collection or evaluation to prevent bias. Data collection was carried out by blinded researchers, including Xuelei Zhou, Xianchun Liu, and Wei Mao. Outcome assessors had no access to allocation information. Statistical analysis was conducted independently by a designated researcher. This design minimizes biases for impartial evaluation.

### Standard anesthesia procedure

All patients followed standard preoperative fasting and fluid restriction protocols. In the operating room, an intravenous line was placed, and compound sodium chloride was infused. Routine monitoring was conducted, and patients were positioned supine and given oxygen at 3 L/min *via* face mask.

After preoperative preparations, each patient received an intravenous injection of 10 mg dexamethasone to reduce the risk of postoperative nausea and vomiting. During anesthesia induction, the R group received intravenous remimazolam (0.3−0.4 mg/kg), and the C group received intravenous propofol (2–3 mg/kg). Both groups then received slow intravenous administration of sufentanil (0.5 µg/kg) (Li et al., 2023) and cisatracurium (0.1−0.2 mg/kg) (Duan et al., 2023).

Following the onset of unconsciousness, manual ventilation was performed *via* a face mask, and orotracheal intubation was conducted using gender-specific reinforced endotracheal tubes—size 6.5 for female patients and size 7.0 for male patients. Post-intubation, patients were connected to a ventilator set to volume-controlled ventilation (VCV) mode. The ventilator parameters were configured with a tidal volume (VT) of 4–8 mL/kg, a respiratory rate (RR) of 12–16 breaths per minute, and end-tidal carbon dioxide pressure (EtCO_2_) maintained between 35–45 mmHg.

During the anesthesia maintenance phase, patients in the R group were administered a continuous intravenous infusion of remimazolam at a rate of 0.6–1 mg/kg/h, while those in the C group received propofol at a rate of 4–10 mg/kg/h (Doi et al., 2020; Kim et al., 2024; Lan et al., 2024; Lee et al., 2024). In addition, both groups were continuously infused with remifentanil at a rate of 0.1–0.2 µg/kg/min. At the conclusion of the surgical procedure, the infusions of remimazolam, propofol, and remifentanil were discontinued. Airway management involved suctioning oral secretions, followed by the administration of intravenous neostigmine (40 µg/kg) and atropine (15 µg/kg) to reverse the effects of cisatracurium (Choi et al., 2016; Zhang et al., 2022). Postoperatively, patients were transferred to the post-anesthesia care unit (PACU), where extubation was performed upon the return of consciousness, spontaneous respiration, and adequate muscle tone. Meanwhile, all surgical procedures in our study were performed by the same experienced surgical team.

Postoperative analgesia followed a standardized surgeon-established protocol applied to all patients: intravenous parecoxib sodium 40 mg q12 h with rescue intravenous tramadol hydrochloride 50–100 mg q6–8 h pro re nata (PRN) for breakthrough pain or inadequate control. Pain was monitored using faces pain scale-revised (FPS-R) targeting ≤ 4, assessed q6 h in the first 24 h after discontinuation of sedatives and q12–24 h thereafter until discharge.

Intraoperative monitoring was conducted to detect and manage hemodynamic instability. If systolic SBP or DBP increased by more than 30% from baseline values, the depth of anesthesia was adjusted accordingly, or urapidil was administered to control the blood pressure. In cases of hypotension, defined as a reduction in SBP or DBP greater than 30% from baseline or an absolute pressure drop below 80/50 mmHg, appropriate doses of ephedrine or norepinephrine were administered to stabilize the patient. For bradycardia (heart rate <45 bpm), 0.3 mg of atropine was administered. In instances of tachycardia (heart rate >110 bpm), esmolol was given as needed to manage the heart rate. Hypoxemia was defined as a SpO2 level below 90%. In the event of hypoxemia, ensure airway patency, administer oxygen, use supplemental or mechanical ventilation if necessary, and conduct blood gas analysis as required. Postoperative metabolic parameters were assessed and rechecked only when clinically indicated.

### Outcome measures

The Sedation-Agitation Scale (SAS) was used to evaluate the level of agitation during emergence from anesthesia (Jo et al., 2019; Kang et al., 2020; Lee et al., 2023). The SAS is a validated seven-point scale ranging from 1 (minimal or no response to noxious stimuli) to 7 (dangerous agitation, such as actively pulling at the endotracheal tube, attempting to remove catheters, or striking at staff) (Riker, Picard & Fraser, 1999). Intermediate scores are defined as follows: Riker SAS assessments were performed by researchers who were trained by a psychiatrist to ensure the accuracy of the evaluations. The SAS was used to measure and record the highest score for each patient. The SAS is categorized as follows:

1: Minimal or no response to noxious stimuli.
2: Arouses to physical stimuli but does not engage in communication.
3: Difficult to arouse, but awakens to verbal stimuli or gentle shaking.
4: Calm and follows commands.
5: Anxious or physically agitated but able to calm with verbal instructions.
6: Requires physical restraint and frequent verbal reminders to remain calm.
7: Actively pulling at the endotracheal tube, attempting to remove catheters, or striking at staff (Riker, Picard & Fraser, 1999).

SAS is considered the standard tool for assessing postoperative EA due to its intuitive nature, ease of use, and ability to quickly identify patients requiring intervention, ensuring postoperative safety and comfort (Barr et al., 2013). Due to its simplicity, wide applicability, and high clinical comparability, the SAS is widely used in the evaluation of agitation following nasal surgery, enabling the assessment of both the incidence and severity of EA (Jo et al., 2019; Kim et al., 2015; Kim et al., 2013; Wegner et al., 2025a). Within the SAS scoring system, a score of 4 indicates that the patient is able to follow commands but may display mild restlessness, while a score of 5 or above indicates significant agitation or anxiety, usually requiring intervention. Consequently, using a score of 4 to define agitation may lead to the misclassification of mild anxiety as agitation, resulting in unnecessary intervention. In contrast, using a score of 5 as the threshold for agitation effectively distinguishes patients who require intervention, preventing both over-intervention and missed diagnoses. This approach demonstrates its high clinical applicability and has been extensively validated in clinical practice.

The SAS assessments were conducted by three researchers (Xuelei Zhou, Xianchun Liu, and Wei Mao) after receiving training under the guidance of psychiatrists. The training included a 2-hour explanation of the scoring criteria, practice with 15 pilot cases, and feedback. Each evaluator also independently scored 10 standard cases. The scoring consistency was tested by having the evaluators independently score 20 real patient cases, with the weighted Kappa coefficient used for assessment. The results showed an average Kappa value of 0.86, indicating good consistency. During the data collection process, scoring drift was checked every two weeks, with evaluators scoring 10 randomly selected new cases. If the difference exceeded one SAS level, a consensus meeting was held to resolve the discrepancies, ensuring consistency.

In cases of severe EA, a rescue dose of intravenous propofol (0.5–1 mg/kg) was administered to prevent serious adverse events (Deng et al., 2022; Zhang et al., 2022). Patients were observed for 30 min in the PACU before being transferred back to the ward, with the SAS score recorded 5 min after extubation.

### Secondary outcome measures

Throughout the anesthesia process, patients’ vital signs and any adverse reactions were meticulously monitored and recorded at specific time points: T0 (baseline, defined as the average of three measurements taken within 10 min after entering the operating room), T1 (immediately before intubation), T2 (5 min after intubation), T3 (at the initiation of surgery), T4 (at the conclusion of surgery), T5 (3 min after extubation), and T6 (10 min after extubation). Variables recorded included surgery duration, emergence time, and adverse reactions such as injection pain, nausea, vomiting, hypertension (mean arterial pressure (MAP) >30% above baseline), and hypotension (MAP >30% below baseline) (Bijker et al., 2007), and bradycardia.

In addition to the intraoperative data, demographic and clinical characteristics of the patients, such as baseline comorbidities, age, gender, weight, height, and ASA classification, were also documented.

### Statistical analysis

Statistical analyses were performed using SPSS version 26.0. The Shapiro–Wilk test was used to assess the normality of continuous variables. Normally distributed data were expressed as mean ± standard deviation (X ± SD), while non-normally distributed data were expressed as median (interquartile range). Parametric data were analyzed using the *t*-test, and non-parametric data were analyzed using the Mann–Whitney U test for comparisons between two independent samples. Categorical variables were compared using the chi-square test or Fisher’s exact test. A *p*-value of less than 0.05 was considered statistically significant.

## Results

**Figure 1:**
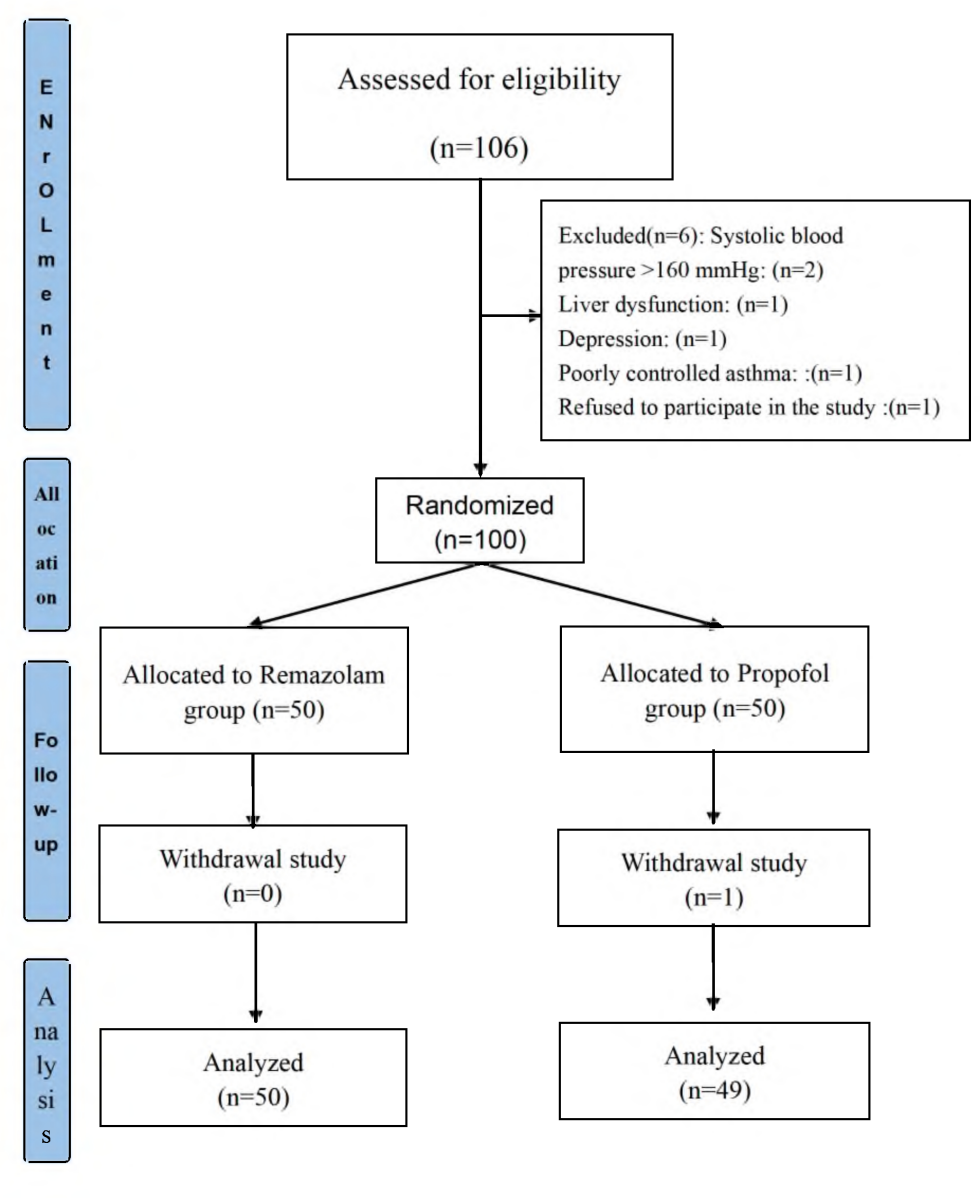
Study flow chart. A total of 106 patients were screened for this study. Six were excluded for not meeting the inclusion criteria, and one withdrew due to a surgery change, leaving 99 participants.

A total of 106 patients were assessed for eligibility in this study. Of these, six patients were excluded due to not meeting the inclusion criteria for the following reasons: two patients had systolic blood pressure exceeding 160 mmHg, one patient had liver dysfunction, one patient had a diagnosis of depression, one patient had poorly controlled asthma, and one patient declined to participate. As a result, 100 patients were successfully enrolled in the study. During the study, one patient was withdrawn due to a change in the surgical procedure, leaving a final cohort of 99 patients who completed the study (Fig. 1).

The baseline characteristics of the study participants are presented in Table 1. No statistically significant differences were observed between the two groups with respect to ASA classification, educational level, type of surgery, comorbid conditions, smoking status, alcohol use, duration of anesthesia, and duration of surgery.

**Table 1:** Baseline demographics of the patients.

	Group R (*n* = 50)	Group C (*n* = 49)	*P*
Age (years)	41 (25.6–56)	43 (24.5–55)	0.902
Gender (male/female)	29/21	31/18	0.741
BMI (kg/m^2^)	22.85 ± 0.35	24.03 ± 0.31	0.106
Hypertension, n (%)	6 (12)	8 (16)	0.537
Diabetes mellitus, n (%)	1 (2)	2 (4)	0.986
Smoker, n (%)	16 (32)	14 (28)	0.711
Drinker, n (%)	6 (12)	4 (8)	0.764
ASA physical status, n (%)			0.831
I	25 (50)	24 (49)	/
II	24 (48)	23 (47)	/
III	1 (2)	2 (4)	/
Level of education, n (%)			0.459
≤primary school, n (%)	5 (10)	6 (12)	/
Junior high school, n (%)	18 (36)	12 (24)	/
≥senior high school, n (%)	27 (44)	31 (63)	/
Septoplasty			0.982
Septal Surgery, n (%)	17 (34)	18 (37)	/
Sinus Surgery, n (%)	7 (14)	6 (12)	/
Septal Surgery+ Sinus Surgery, n (%)	19 (38)	19 (39)	/
Combined Surgery, n (%)	7 (14)	6 (12)	/
Duration of anesthesia (min)	95.34 ± 26.21	103.98 ± 27.43	0.113
Duration of surgery (min)	75.76 ± 22.62	82.02 ± 19.58	0.144

**Notes.**

Values are presented as median (Q1, Q3), number, mean ± SD, or number (%). Categorical variables were compared using the Chi-square test or Fisher’s exact test. For normally distributed data, an independent samples *t*-test was used, while the Mann–Whitney U test was applied for non-normally distributed data.

R group Remimazolam group C group Propofol group BMI Body Mass Index ASA American Society of Anesthesiologists classification

**Figure 2:**
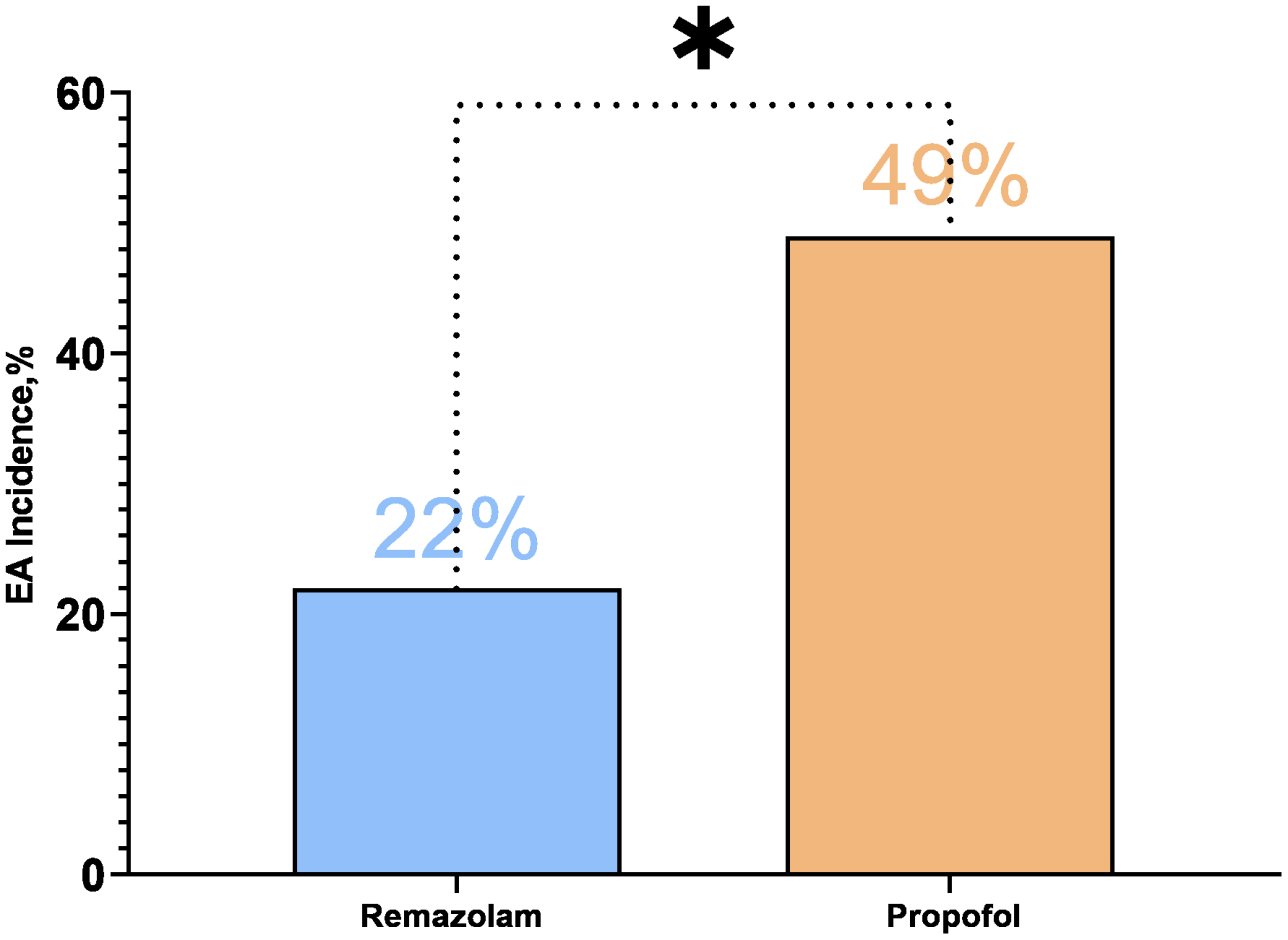
Incidence of EA in both groups.

The incidence of EA was significantly lower in the R group compared to the C group. Specifically, 22% of patients in the R group (11 out of 50) exhibited EA, compared to 49% in the C group (24 out of 49) (22% *vs.* 49%; risk difference −27% (95% CI [−50% to −0.3%]); Relative Risk 0.45 (95% CI [0.25–0.81]); *P* = 0.005). The results indicate a statistically significant reduction in EA in the R group (Fig. 2).

These data indicate that the incidence of EA in the R group was reduced by 55% compared to the C group, suggesting that remimazolam has a significant advantage in minimizing EA. Additionally, the immediate SAS scores further support this finding. The immediate SAS scores were significantly lower in the R group than in the C group (*P* = 0.012). Moreover, the proportion of patients achieving a six points score was significantly lower in the R group compared to the C group (6% *vs.* 20%, *P* = 0.034), while the proportion of patients achieving a Four points score was significantly higher in the R group (62% *vs.* 39%, *P* = 0.021). All these differences were statistically significant (Table 2). Additionally, EA in all patients subsided within 5 min, with no patients requiring re-sedation.

**Table 2:** Comparison of SAS scores between groups two.

	Group R (*n* = 50)	Group C (*n* = 49)	*P*
SAS			
0 min	4 (4–4)	4 (4–6)	0.012
5 min	4 (4–4)	4 (4–4)	0.106
One point	0	0	/
Two points	0	0	/
Three points	8	6	0.592
Four points	31	19	0.021
Five points	4	4	1
Six points	3	10	0.034
Seven points	4	10	0.076

**Notes.**

R group Remimazolam group C group Propofol group SAS Riker Sedation-Agitation Scale

**Figure 3:**
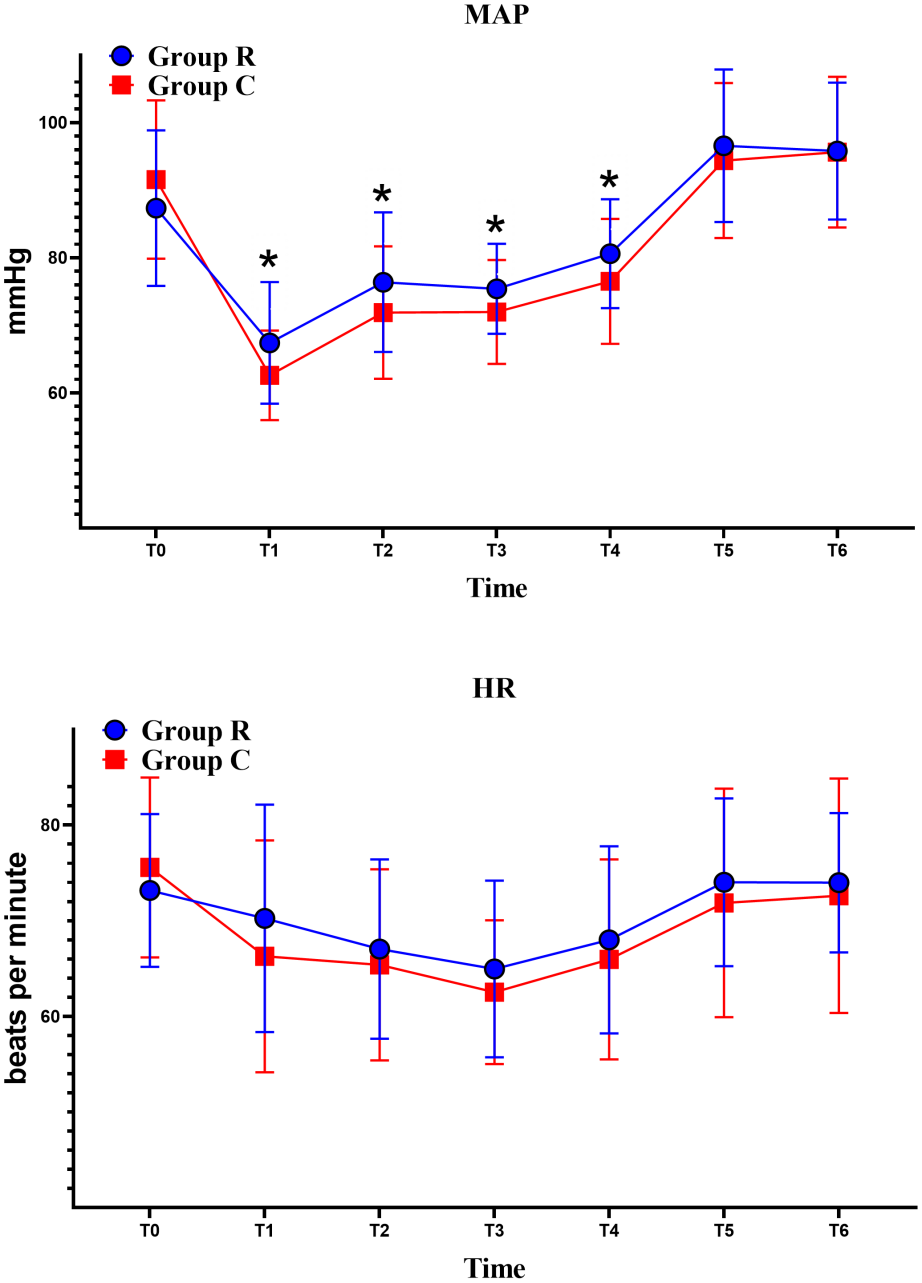
Hemodynamic changes in both groups. R group: Remimazolam group; C group: Propofol group; MAP: Mean Arterial Pressure; HR: Heart Rate. T0 (Baseline after admission), T1 (Before intubation), T2 (5 min after intubation), T3 (At the start of surgery), T4 (At the end of surgery), T5 (3 min after extubation), and T6 (10 min after extubation). Comparison of MAP between the two groups, the MAP value was higher in the R group at the T1–T4 time point and the comparison was statistically significant (*P < 0.05).

At time points T1, T2, T3, and T4, the MAP in the R group was higher than in the C group, with statistically significant differences. Although the heart rate (HR) in the R group was higher than in the C group after the start of the surgery, this difference was not statistically significant (Fig. 3).

The results showed that the emergence time in the R group was significantly longer than that in the C group (8.24 ± 2.65 *vs.* 6.86 ± 2.08, *P* = 0.005). Similarly, the extubation time was also significantly longer in the R group compared to the C group (8.62 ± 2.59 *vs.* 7.28 ± 1.98, *P* = 0.005). However, the R group demonstrated a significant advantage in terms of the incidence of adverse effects. Specifically, the incidence of injection pain was dramatically lower in the R group than in the C group (2% *vs.* 41%, *P* < 0.001), and the incidence of hypotension was also markedly reduced (18% *vs.* 53%, *P* < 0.001). Although the incidence of bradycardia was lower in the R group compared to the C group (0% *vs.* 8%, *P* = 0.121), this difference was not statistically significant. There was no significant difference observed in postoperative wound bleeding between the two groups. Additionally, no patients in either group experienced postoperative nausea or vomiting (Table 3). No adverse reactions such as hypoxemia were observed in the patients in this study.

**Table 3:** Adverse reaction.

	Group R	Group C	*P*
Awakening time (min)	8.24 ± 2.65	6.86 ± 2.08	0.005
Time to extubation (min)	8.62 ± 2.59	7.28 ± 1.98	0.005
Injection pain, n (%)	1 (2)	20 (41)	<0.001
Hypotension, n (%)	9 (18)	26 (53)	<0.001
Bradycardia, n (%)	0	4 (8)	0.121
Wound bleeding, n (%)	0	1 (2)	0.992
Nausea and Vomiting, n (%)	0	0	/

**Notes.**

Values are presented as median (Q1, Q3), number, mean ± SD, or number (%). Categorical variables were compared using the Chi-square test or Fisher’s exact test. For normally distributed data, an independent samples *t*-test was used.

R group Remimazolam group C group Propofol group

## Discussion

This randomized controlled trial demonstrates that remimazolam significantly reduces the incidence of EA after nasal surgery compared to propofol, providing new evidence for its efficacy in this high-risk surgical context. Additionally, remimazolam was associated with better hemodynamic stability and less injection pain, while rates of bradycardia, postoperative wound bleeding, and nausea and vomiting were similar between groups.

EA is consistently reported after otolaryngologic procedures, and nasal surgery carries a particularly high baseline risk, with incidence approaching 50–55% in prior studies (Demir & Yuzkat, 2018; Lee et al., 2019; Yu et al., 2010). A concise, evidence-based explanation is that nasal surgery concentrates several potent emergence-phase triggers: (1) local tissue trauma and postoperative discomfort, (2) upper-airway irritation from blood and secretions, and (3) functional nasal obstruction due to swelling and/or packing, which can generate a pronounced sensation of dyspnea or suffocation even when objective airway patency is adequate (Eckenhoff, Kneale & Dripps, 1961). These factors plausibly amplify sympathetic arousal and distress during early recovery and help contextualize the 49% EA incidence observed in our propofol group.

It is widely acknowledged in the academic community that EA is associated with the excitation phase following anesthesia recovery. As consciousness gradually returns, patients may exhibit an exaggerated response to both internal and external stimuli, characterized by excessive arousal (Brown, Lydic & Schiff, 2010). Neurophysiological hypotheses further propose that asynchronous recovery across neural circuits may contribute to this mismatch and predispose patients to agitation (Bonhomme et al., 2012). In nasal surgery—where airway-related discomfort and perceived obstruction are prominent—these emergence-phase dynamics are likely to be especially relevant. Within this framework, remimazolam’s pharmacology provides a clear and clinically meaningful link to EA mechanisms. Remimazolam is an ultra-short-acting benzodiazepine that positively modulates GABA-A receptors, enhancing inhibitory neurotransmission and attenuating neuronal excitability. If EA reflects excessive arousal and heightened stimulus reactivity during emergence, strengthening inhibitory tone *via* GABAergic mechanisms may mitigate hyperarousal and facilitate better behavioral control at a time when airway irritation and obstruction-related discomfort are intense. In addition, remimazolam is primarily metabolized by non-specific esterases, a feature associated with predictable offset and limited accumulation across individuals (Schüttler et al., 2020; Sneyd, Gambus & Rigby-Jones, 2021; Stöhr et al., 2021). Predictable recovery kinetics may support a smoother emergence trajectory—an advantage in nasal surgery, where distressing sensations can otherwise trigger agitation.

Our results are consistent with this interpretation. Emergence time was longer in the remimazolam group, and rapid awakening has been identified as a risk factor for EA in some clinical contexts (Moore & Anghelescu, 2017). A more gradual transition to wakefulness may reduce the likelihood that intense airway- and discomfort-related stimuli coincide with partial cognitive recovery. By contrast, propofol—although often associated with faster awakening—may permit a rapid return of consciousness at a time when nasal packing, blood/secretions, and airway irritation remain prominent, potentially increasing agitation risk in susceptible patients (Sahinovic, Struys & Absalom, 2018; Schüttler et al., 2020). While the relationship between benzodiazepines and EA has been debated in some studies (Chandler et al., 2013; Zhang et al., 2013), multiple studies evaluating remimazolam have consistently reported reduced emergence-related agitation across various surgical populations. Pediatric studies have shown lower rates of EA after laparoscopic surgery and tonsillectomy when remimazolam was administered as a bolus or infusion near the end of anesthesia (Cai et al., 2024; Yang et al., 2022) and similar results have been observed in elderly patients undergoing hip replacement (Duan et al., 2023). These findings align with our results and support the potential of remimazolam in mitigating emergence-related agitation, especially when emergence is triggered by strong noxious or airway stimuli.

SAS is widely used in adults and, as a common tool for assessing EA, has been validated for good inter-rater reliability, as well as high reliability and effectiveness (Kim et al., 2013; Lepousé et al., 2006; Namigar et al., 2017; Riker et al., 2001; Riker, Picard & Fraser, 1999). First, SAS is easy to use, particularly for rapid assessment in emergency situations; second, it shows high reliability and a good correlation with the Harris and Ramsay scales (Riker et al., 2001). Furthermore, SAS categorizes EA into multiple levels, providing more clinical information without compromising its effectiveness and reliability (Riker, Picard & Fraser, 1999).

In addition to lowering EA incidence, remimazolam favorably shifted agitation severity: fewer patients reached very high SAS levels, and a higher proportion were calm and responsive to commands (SAS = 4). Consistently, fewer patients in the R group had SAS immediate scores of six and seven compared with the control group; although not all between-group differences reached statistical significance, this distributional shift suggests a potential reduction in severe agitation and an improvement in emergence quality.

Safety outcomes were also clinically relevant. Remimazolam was associated with fewer hypotensive events than propofol, consistent with prior evidence suggesting hemodynamic advantages of remimazolam during anesthesia (Chae et al., 2022; Choi et al., 2022; Wang et al., 2022). Injection pain was also markedly reduced. No serious adverse events were observed, and common postoperative adverse events were similar between groups.

There are some limitations in this study. First, the study did not assess short-term or long-term neurocognitive changes, leaving the potential impact of remimazolam on neurocognitive outcomes unexplored. Second, there is no standardized assessment criterion for EA, and variations in criteria may lead to differences in reported incidence rates (Lee & Sung, 2020), affecting the comparability of our results with other studies. Additionally, postoperative pain scores were not collected in this study, and the potential rebound pain associated with remifentanil may have influenced the results. Finally, the small sample size and single-center design may limit the generalizability of the results.

## Conclusions

Remimazolam holds significant clinical application value and potential in surgeries with a high incidence of EA.

##  Supplemental Information

10.7717/peerj.21018/supp-1 Supplemental Information 1 CONSORT 2010 Checklist

10.7717/peerj.21018/supp-2 Supplemental Information 2 Study protocol

10.7717/peerj.21018/supp-3 Supplemental Information 3 Original data

## Additional Information and Declarations

### Data Availability

The following information was supplied regarding data availability: The raw data is available in the Supplemental File.

### Clinical Trial Registration

The following information was supplied regarding Clinical Trial registration: ChiCTR2400085806.
