# Supplementary material for: The effects of remimazolam on emergence agitation in patients undergoing nasal surgery: a clinical randomized controlled trial
Source: PeerJ. 2026 Mar 19;14:e21018. doi: 10.7717/peerj.21018 (PMC13006008; doi:10.7717/peerj.21018)
Supplement: Supplemental Information 2 [file peerj-14-21018-s002.docx]

**The study protocol has been registered on the China Clinical Trial Registry (www.chictr.org.cn) with the registration number ChiCTR2400085806. You can view the study protocol online via the following link:** <https://www.chictr.org.cn/showproj.html?proj=231013>
